# Analytical and computational studies predict negligible risk of cell death from eddy generation off flat surfaces in cell culture flow systems

**DOI:** 10.3389/fbioe.2024.1340653

**Published:** 2024-08-07

**Authors:** Elliot J. Morley, Claire L. Brockett, Stefaan W. Verbruggen

**Affiliations:** ^1^ Department of Mechanical Engineering, University of Sheffield, Sheffield, United Kingdom; ^2^ INSIGNEO Institute for in silico Medicine, University of Sheffield, Sheffield, United Kingdom; ^3^ Centre for Predictive in vitro Models and Centre for Bioengineering, School of Engineering and Materials Science, Queen Mary University of London, London, United Kingdom; ^4^ Digital Environment Research Institute, Queen Mary University of London, London, United Kingdom

**Keywords:** cell culture therapy, fluid shear stress, eddy formation, microfluidics, T cell bioreactor, Reynolds number

## Abstract

Cell-based therapies represent the current frontier of biomedical innovations, with the technologies required underpinning treatments as broad as CAR-T cell therapies, stem cell treatments, genetic therapies and mRNA manufacture. A key bottleneck in the manufacturing process for each of these lies in the expansion of cells within a bioreactor vessel, requiring by far the greatest share of time for what are often time-critical therapies. While various designs, culture feeding and mixing methods are employed in these bioreactors, a common concern among manufacturers and researchers lies in whether shear stresses generated by culture media flow will damage cells and inhibit expansion. This study develops an analytical tool to link macro-scale measures of flow to risk of cell death using relationships with eddy size and dissipation rates, from eddies generated off flat surfaces. This analytical tool was then employed using computational fluid dynamics (CFD) to replicate a range of generic bioreactor geometries and flow conditions. We found that no combination of flow condition or design parameter was predicted by the tool to cause cell death within eddies, indicating negligible risk of cell death due to eddy formation within cell culture systems. While this requires experimental validation, and does not apply when cells are expanded using microcarriers, this tool nonetheless provides reassurance and accessible prediction of bioreactor design parameters that could result in cell death. Finally, our findings show that bioreactor design can be tailored such that the shear stress stimulation of cells can be selectively altered through small changes in flow rate.

## 1 Introduction

The current frontier of biomedical therapies lies a range of novel patient-specific molecular tools, from personalised mRNA vaccines, to gene therapies and chimeric antigen receptor T cell (CAR-T cell) treatments. Indicating the breadth of new opportunities, as of April 2022 there were 2756 active clinical trials for various cell therapies (Saez-Ibañez et al., 2022). The manufacturing methods and capacity for these cell therapies must be optimised and scaled to support the volume of both trial and marketed products. To illustrate the scale of the challenge, industry leaders estimate that the 6 currently available CAR-T therapies are reaching only 2% of the potential target population, partly due to this lack of scale (BioInformant, 2023). Therefore, a ∼50-fold increase in output is required just to appropriately support the current therapies, which are extremely expensive but potentially curative (Melenhorst et al., 2022), before accounting for the output required for clinical trials and any therapies approved in the future.

A key step in bringing each of these treatments to the clinic involves rapid expansion of a cell population, often from the specific patient as in autologous therapies. While up to 40 separate operations may be performed to manufacture a single therapy, with 120 separate consumables, arguably the most critical component is the expansion vessel or bioreactor. This expansion comprises the largest amount (>90%) of the time involved in delivering these treatments, which can be time-critical in the case of cancer patients required CAR-T cell therapies. Therefore, reliable and controlled expansion presents a significant engineering challenge for the delivery of these treatments.

Cell death during this expansion phase is a significant risk for cell-based therapies, with multiple risk factors and causes. Almost all bioreactor models comprise a sterile chamber with a carefully controlled environment in which cell numbers can multiply and expand. In the case of CAR-T therapy manufacture, the expanded T-cells are in suspension in cell culture media, as opposed to being adherent cells requiring a surface. Cell culture media for expansion can be fed in through multiple different ways, including batch, fed-batch and continuous perfusion, with each of these methods imparting some form of fluid shear stress onto the cells. In some models that utilise continuous perfusion, higher flows are actively applied to increase mixing and space for expansion. However, this gives rise to a common concern for many researchers and manufacturers that shear stress caused by flow can exceed membrane strength and rip the cells apart within bioreactors, particularly through the formation of eddies. Eddies within the system will decay through the phenomenon known as the energy cascade, the progressive breakdown of larger eddies into smaller eddies. This terminates at the Kolmogorov length scale, where viscous forces become significant and energy is dissipated as heat (Kolmogorov, 1968). All eddies experience this decay, no matter the method of formation; from a turbine, from a flat surface, or through random turbulence (Croughan, 1988; Van’t Riet and Smith, 1975). A number of previous experimental studies have investigated the effects of hydrodynamic forces on cells cultured on microcarriers, suspended surfaces for adherent cells to grow on (7,8), with these studies finding that cell death could occur when eddy size fell below the size of the microcarrier (Croughan, 1988). Despite this finding, at present there is no simple or standard method to experimentally predict eddy size, and therefore risk of cell death, within a specific bioreactor design. Indeed, at predicting the likelihood of strong eddy formation within cell culture systems, fed-batch bioreactors and mixing regimes remains challenging.

The objective of this study was, firstly, to mathematically link eddy formation with risk of cell death using a predictive analytical modelling approach. No other causes of non-programmed cell death are considered in this approach. Building upon this, series of geometries representing standard bioreactor and fluidic systems were modelled computationally. Various design changes via baffles to alter flow patterns were also modelled, determining their effect on eddy formation off flat surfaces and resulting wall shear stresses (WSS).

## 2 Materials and methods

### 2.1 Analytical modelling of eddy formation and resulting risk of cell damage from fluid shear stress

All flow systems generate some degree of turbulence, and therefore cause formation of eddies. Some bioreactors actively introduce turbulence, and therefore shear, to mix batches, while still others apply turbulence to mechanically stimulate cells.

These turbulence inducing designs should consider the Kolmogorov microscale and the effect on cells. As eddies form in turbulent flow, energy is transferred from larger to smaller eddies. The smallest eddy, where energy is dissipated as heat as opposed to transferred to another eddy, is known as the Kolmogorov microscale (η) and is evaluated as:

$$\eta ={\left(\frac{\nu^{3}}{\epsilon }\right)}^{\frac{1}{4}}$$

where ν is the kinematic viscosity and ϵ represents average rate of dissipation of turbulent kinetic energy per unit mass.

At cellular length scales, turbulence is both chaotic and intermittent. The resultant large velocity gradients produced in these small eddies can result in high shear, which may result in cell damage and death. Nienow et al. noted that if the entity diameter (in this case, cell diameter) is < *η*, it will not be damaged (Nienow et al., 2013). This makes intuitive sense, as an entity the size of an eddy will experience opposing velocity gradients on the surface, and therefore very high shear stress. This finding is noted repeatedly in the literature (Papoutsakis, 1991; Millward et al., 1994; Chalmers, 2015; Chalmers, 2021), with *η* ≤ 20 μm generally being considered as dangerous to the cell, depending on cell diameter.

Millward et al. provide a bioreactor design and experimental data indicating rapid decline in cell viability at Kolmogorov scales roughly equal to cell diameter (Millward et al., 1994). An interesting point is made by Chalmers (Chalmers, 2021) in his synthesis of existing literature that values of ϵ around 10^6^ correlate with immediate cell lysis, which is equivalent to wall shear stresses (WSS) higher than 5 Pa (50 dyn/cm^2^). Both of these papers are in agreement with the content authored by Nienow et al. (Nienow et al., 2013).

A useful approximation can be made using the Kolmogorov microscale, substituting and estimation of the energy dissipation rate by approximating eddy time scale with *L/U*:

$$\epsilon ∼\frac{UU}{L/U}∼\frac{U^{3}}{L}$$

where *L* is the characteristic length and *U* is the flow velocity. By combining, the previous equation:

$$\eta ={\left(\frac{\nu^{3}L}{U^{3}}\right)}^{\frac{1}{4}}$$

This allowed us to link eddy formation and Reynolds number to risk of cell death, giving an estimated ratio of smallest to largest eddy sizes, as follows.

$$\frac{L}{\eta }∼{\left(\frac{UL}{\nu }\right)}^{\frac{3}{4}}={Re}^{\frac{3}{4}}$$

where *Re* signifies the Reynolds number.

### 2.2 Computational modelling of eddy formation and resulting shear stress within a generic bioreactor system

As the flow in a complex geometry, such as that found in a bioreactor, is too complicated to solve analytically, a computational modelling approach was taken. Computational fluid dynamics (CFD) is a standard engineering technique commonly used in biomedical engineering, both for parametric design of devices (such as bioreactors) and for directly studying mechanical stimulation of cells (Dolan et al., 2018). Using ANSYS Fluent 2021 R1, a series of computational fluid dynamics (CFD) simulations were generated to determine the effects of various generic bioreactor design changes on flow behaviour, and ultimately infer the effect of changes on cells. The geometry mimics modified Corning cell culture flasks. Fluent solves the Navier Stokes equations in a numerical fashion. Specifically, Reynolds Averaged Navier Stokes (RANS) equations are used over the unmodified Navier Stokes equations to model the time-independent flow through the inclusion of Reynolds stresses.

The Reynolds stresses were solved for using an appropriate turbulence model. As the domain modelled includes a form of jet flow, the kω-SST turbulence model was applied in this study. This is the case even if a low Reynolds number flow condition was used, as the geometries were designed to induce some form of mixing alongside producing separating flow at the inlet. This turbulence modelling approach is commonly used to investigate the effect of design features such as baffles in the literature (Saim et al., 2013; Pal et al., 2016; Tahmasebi et al., 2020), finding that this model tends to outperform others in steady-state prediction of mixing. This is likely because alternative models will not capture the near-wall effects of a baffled internal flow (Murthy and Joshi, 2008).

**FIGURE 1:**
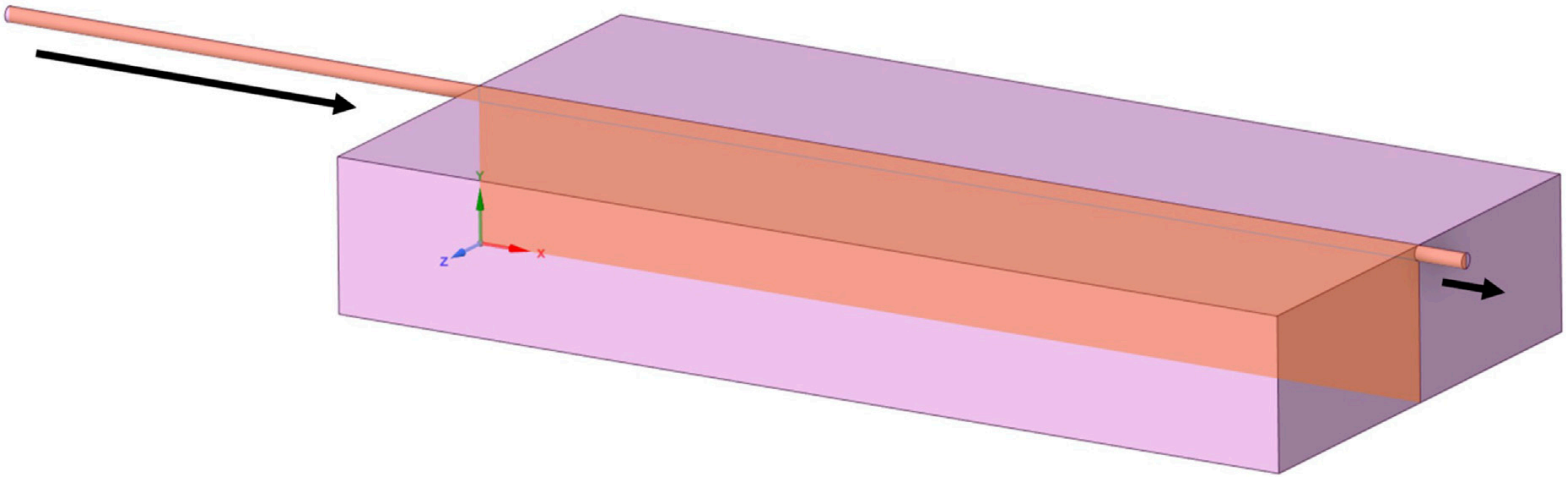
A generic bioreactor geometry for continuous perfusion, with slice representing 2D simplification for modelling and arrows indicating inlets and outlets.

Each geometry was based on a generic cuboidal bioreactor, and thus modelled using a 2D slice incorporating the inlet and outlet (see Figure 1). Spanwise behaviour was assumed to be negligible. Flow was assumed to be incompressible, with no-slip boundary conditions at walls. The model was assumed to be an adiabatic and isothermal system, at 37°C. Cell culture media properties were assumed to be equivalent to water, with density *ρ* = 994 kg/m^3^ and dynamic viscosity *μ* = 0.0007191 Pa.s.

Given that the models assumed continuous perfusion, a velocity inlet boundary condition could be paired with a pressure and mass flow outlet boundary condition. Industry media feed and/or fill rates can vary from less than 1 mL/min to upwards of 100 mL/min, and thus 4 sets of boundary conditions were determined to consider four orders of magnitude of flow rates from 0.1 mL/min to 100 mL/min. The exact boundary conditions can be found in Table 1.

**TABLE 1:** Boundary conditions applied to each geometry during modelling procedure.

Flow rate (mL/min)	Inlet velocity (m/s)	Inlet turbulence intensity (%)	Outlet gauge pressure (Pa)	Outlet mass flow rate (kg/s)
0.1	5.8946 × 10^−5^	1	0	4.158 × 10^−7^
1	5.8946 × 10^−4^	1	0	4.158 × 10^−6^
10	5.8946 × 10^−3^	1	0	4.158 × 10^−5^
100	5.8946 × 10^−2^	1	0	4.158 × 10^−4^

**FIGURE 2:**
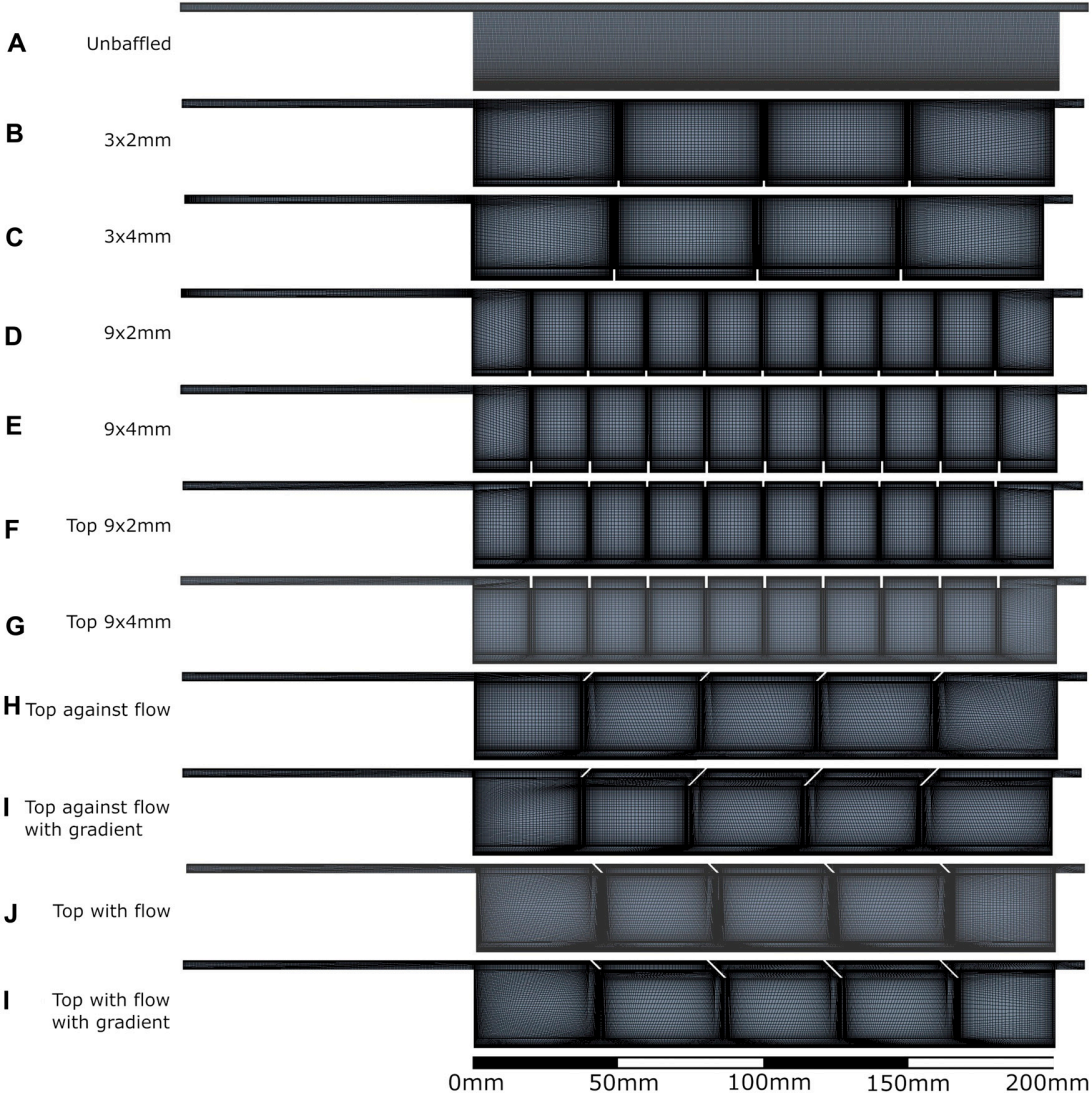
All bioreactor designs and meshes applied in the computational modelling process. **A**: unbaffed design, **B**: 3 baffles of 2 mm height on the base, **C**: 3 baffles of 4 mm height on the base, **D**: 9 baffles of 2 mm height on the base, **E**: 9 baffles of 4 mm height on the base, **F**: 9 baffles of 2 mm height on the top surface, **G**: 9 baffles of 4 mm height on the top surface, **H**: 4 baffles of 4 mm height angled at 45^ into the flow, **I**: 1 baffle of 4mm height followed by 3 baffles of 8 mm height, all angled at 45^ into the flow, **J**: 4 baffles of 4 mm height angled at 45^ away from the flow, **K**: 1 baffle of 4 mm height followed by 3 baffles of 8 mm height, all angled at 45^ away from the flow.

Density of meshes created for each geometry tested are shown in Figure 2, with all meshes checked for convergence (see Supplementary Material). Meshes were composed of quadratic quadrilateral elements in a semi-structured fashion.

## 3 Results

### 3.1 Analytical model links eddy formation to risk of cell death

**FIGURE 3:**
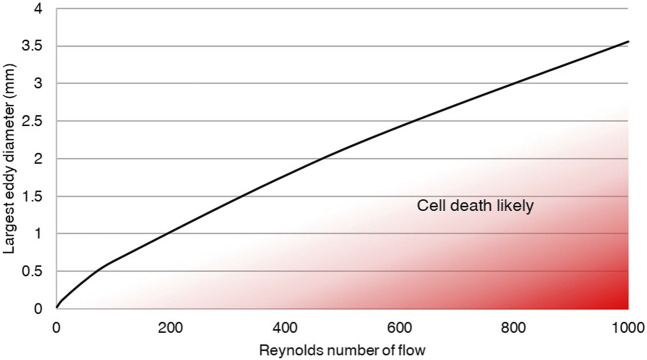
Eddies must be kept large to prevent cell damage through eddy dissipation. The analytical relationship developed between eddy size and Reynolds number demonstrates the need to ensure large eddy sizes relative to cells in order to prevent cell death. The plotted line indicates the predicted flow and maximum eddy size within a fluid at which turbulent decay will produce eddies on the scale of cells, and therefore risk cell death.

Taking the assumption that Kolmogorov microscale, *η*, must be greater than a cell diameter, allowed calculation of the risk of cell death at various maximum eddy diameters and Reynolds numbers, assuming *η* ≤ 20 μm. This risk relationship, calculated in Eq. 4, is shown visually in Figure 3, with graph data included in Supplementary Material. If maximum measured eddy size is below the curve, dissipation will result in cell-sized eddies, resulting in extreme shear on the cells and likely cell death.

### 3.2 Computational models predict effects of bioreactor design on eddy formation

**FIGURE 4:**
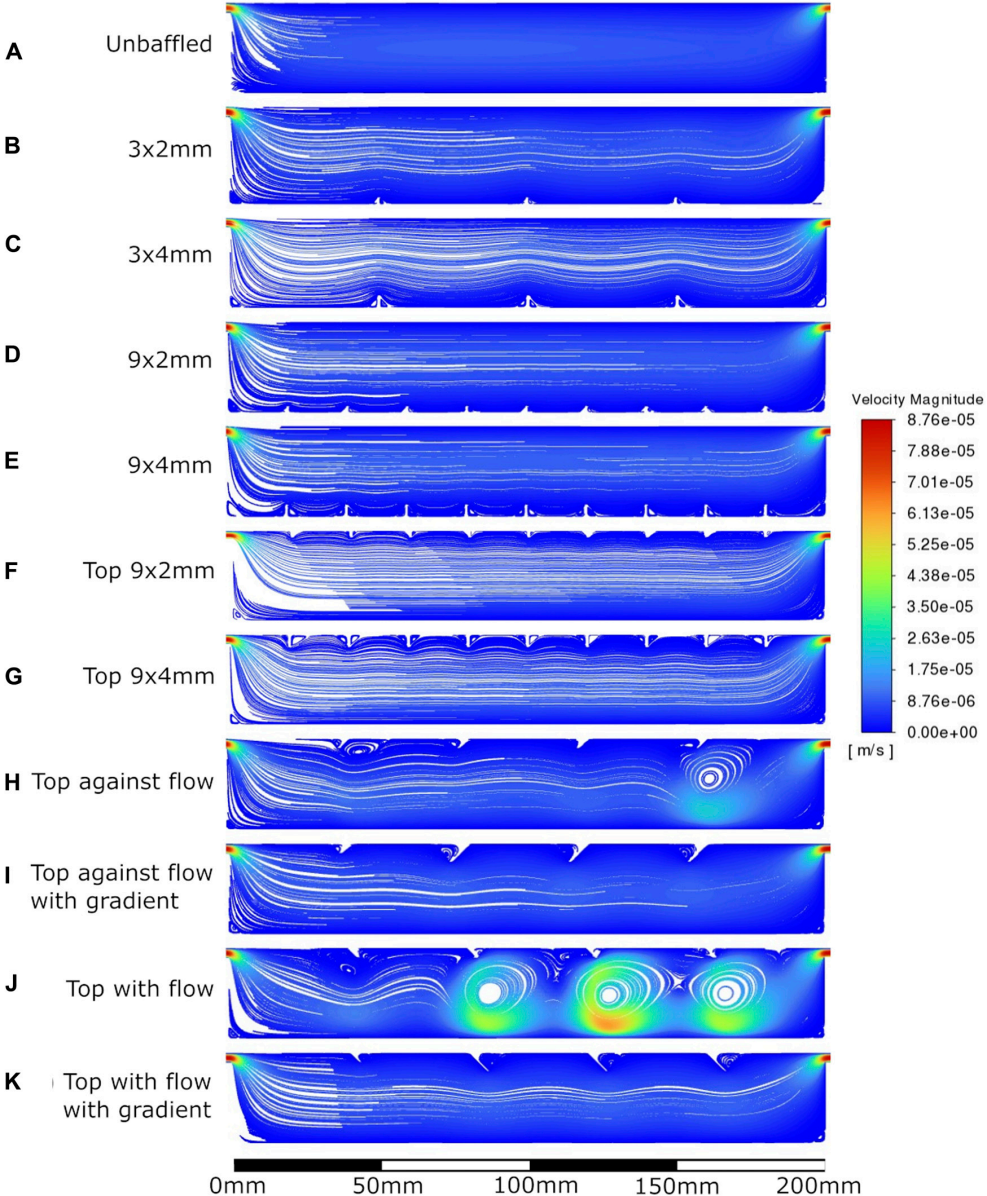
Velocity streamlines at 0.1 mL/min flow rate. **A**: unbaffed design, **B**: 3 baffles of 2 mm height on the base, **C**: 3 baffles of 4 mm height on the base, **D**: 9 baffles of 2 mm height on the base, **E**: 9 baffles of 4 mm height on the base, **F**: 9 baffles of 2 mm height on the top surface, **G**: 9 baffles of 4 mm height on the top surface, **H**: 4 baffles of 4 mm height angled at 45^ into the flow, **I**: 1 baffle of 4 mm height followed by 3 baffles of 8 mm height, all angled at 45^ into the flow, **J**: 4 baffles of 4 mm height angled at 45^ away from the flow, **K**: 1 baffle of 4 mm height followed by 3 baffles of 8 mm height, all angled at 45^ away from the flow.

**FIGURE 5:**
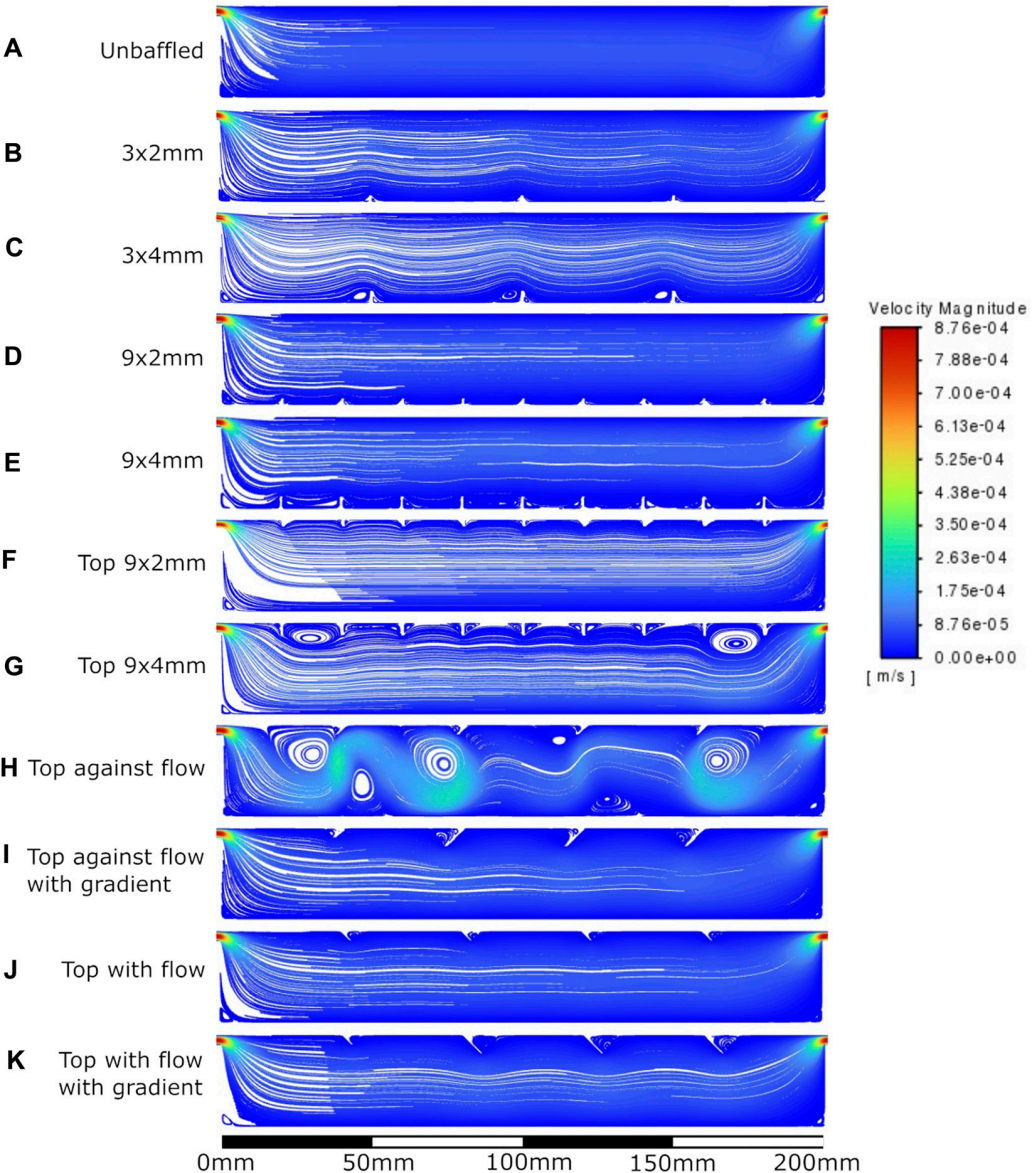
Velocity streamlines at 1 mL/min flow rate. **A**: unbaffed design, **B**: 3 baffles of 2 mm height on the base, **C**: 3 baffles of 4 mm height on the base, **D**: 9 baffles of 2 mm height on the base, **E**: 9 baffles of 4 mm height on the base, **F**: 9 baffles of 2 mm height on the top surface, **G**: 9 baffles of 4 mm height on the top surface, **H**: 4 baffles of 4 mm height angled at 45^ into the flow, **I**: 1 baffle of 4 mm height followed by 3 baffles of 8 mm height, all angled at 45^ into the flow, **J**: 4 baffles of 4 mm height angled at 45^ away from the flow, **K**: 1 baffle of 4 mm height followed by 3 baffles of 8 mm height, all angled at 45^ away from the flow.

**FIGURE 6:**
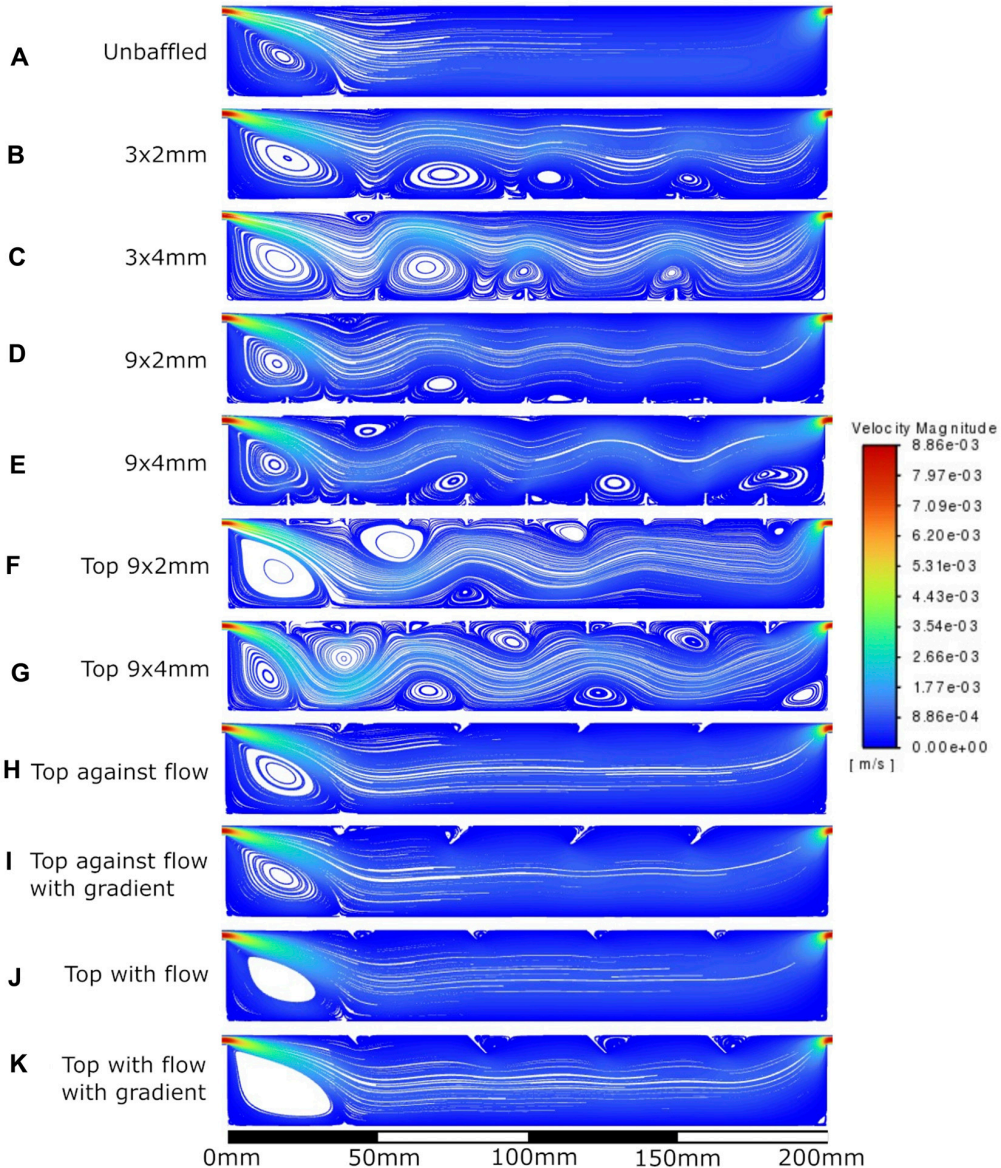
Velocity streamlines at 10 mL/min flow rate. **A**: unbaffed design, **B**: 3 baffles of 2 mm height on the base, **C**: 3 baffles of 4 mm height on the base, **D**: 9 baffles of 2 mm height on the base, **E**: 9 baffles of 4 mm height on the base, **F**: 9 baffles of 2 mm height on the top surface, **G**: 9 baffles of 4 mm height on the top surface, **H**: 4 baffles of 4 mm height angled at 45^ into the flow, **I**: 1 baffle of 4 mm height followed by 3 baffles of 8 mm height, all angled at 45^ into the flow, **J**: 4 baffles of 4 mm height angled at 45^ away from the flow, **K**: 1 baffle of 4 mm height followed by 3 baffles of 8 mm height, all angled at 45^ away from the flow.

**FIGURE 7:**
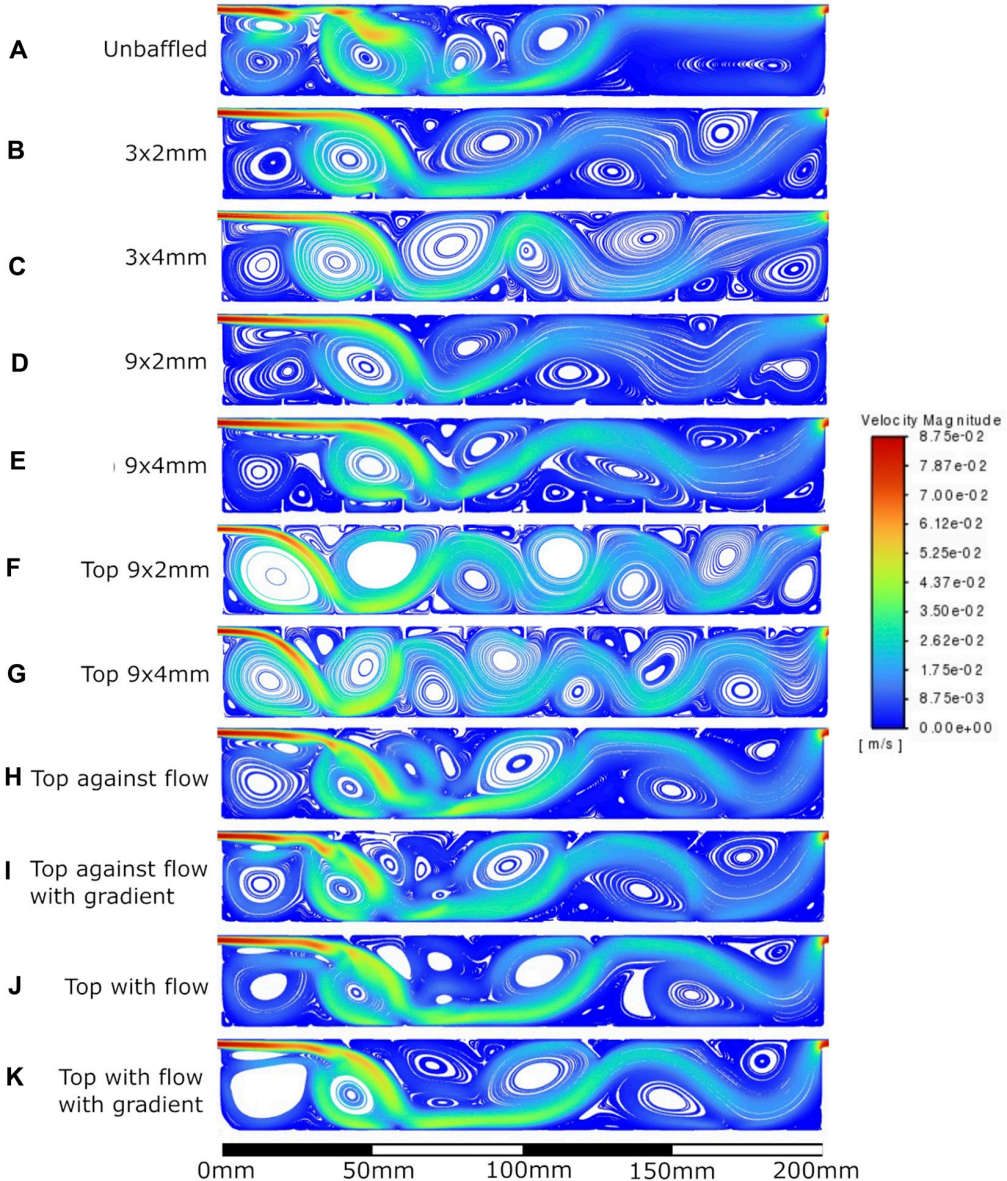
Velocity streamlines at 100 mL/min flow rate. **A**: unbaffed design, **B**: 3 baffles of 2 mm height on the base, **C**: 3 baffles of 4 mm height on the base, **D**: 9 baffles of 2 mm height on the base, **E**: 9 baffles of 4 mm height on the base, **F**: 9 baffles of 2 mm height on the top surface, **G**: 9 baffles of 4 mm height on the top surface, **H**: 4 baffles of 4 mm height angled at 45^ into the flow, **I**: 1 baffle of 4 mm height followed by 3 baffles of 8mm height, all angled at 45^ into the flow, **J**: 4 baffles of 4 mm height angled at 45^ away from the flow, **K**: 1 baffle of 4 mm height followed by 3 baffles of 8 mm height, all angled at 45^ away from the flow.

The velocity fields observed within each model geometry are displayed as streamlines in Figures 4–7, for increasing flow rates from 0.1–100 mL/min, respectively. These data have been collected across 11 separate models and 4 separate flow conditions, totalling 44 sets of data. Patterns of eddy formation generated in these various designs are shown in Table 2.

**TABLE 2:** Results of largest eddy measurements of models, the estimated smallest eddy using the Kolmogorov microscale, and the ratio between smallest eddy and cell diameters (assuming cell diameter = 0.02 mm). Largest eddy on bottom surface of model measured. Not shown is 0.1 mL/min flow condition, as Stokes flow is expected.

Flow rate	Inlet reyonlds number	Largest:smallest eddy ratio	Condition	Largest eddy	Smallest eddy	Smallest eddy:cell ratio
1	2.7	2.11	Unbaffled	3.1	1.5	73.6
			3 × 2	2.9	1.4	68.8
			3 × 4	6.3	3.0	149.6
			9 × 2	3.0	1.4	71.2
			9 × 4	4.2	2.0	99.7
			Top 9 × 2	3.1	1.5	73.6
			Top 9 × 4	19.9	9.4	472.4
			Top against flow	25.4	12.1	602.9
			Top against flow with gradient	6.0	2.8	142.4
			Top with flow	4.0	1.9	95.0
			Top with flow with gradient	3.7	1.8	87.8
10	27	11.84	Unbaffled	40.6	3.4	171.4
			3 × 2	44.4	3.7	187.4
			3 × 4	37.2	3.1	157.0
			9 × 2	31.3	2.6	132.1
			9 × 4	29.4	2.5	124.1
			Top 9 × 2	37.7	3.2	159.1
			Top 9 × 4	20.8	1.8	87.8
			Top against flow	41.3	3.5	174.3
			Top against flow with gradient	42.0	3.5	177.3
			Top with flow	42.6	3.6	179.8
			Top with flow with gradient	41.8	3.5	176.5
10	270	66.61	Unbaffled	34.6	0.5	26.0
			3 × 2	28.3	0.4	21.2
			3 × 4	31.8	0.5	23.9
			9 × 2	38.7	0.6	29.1
			9 × 4	17.8	0.3	13.4
			Top 9 × 2	34.4	0.5	25.8
			Top 9 × 4	30.5	0.5	22.9
			Top against flow	27.1	0.4	20.3
			Top against flow with gradient	34.2	0.5	25.7
			Top with flow	34.9	0.5	26.2
			Top with flow with gradient	31.7	0.5	23.8

**FIGURE 8:**
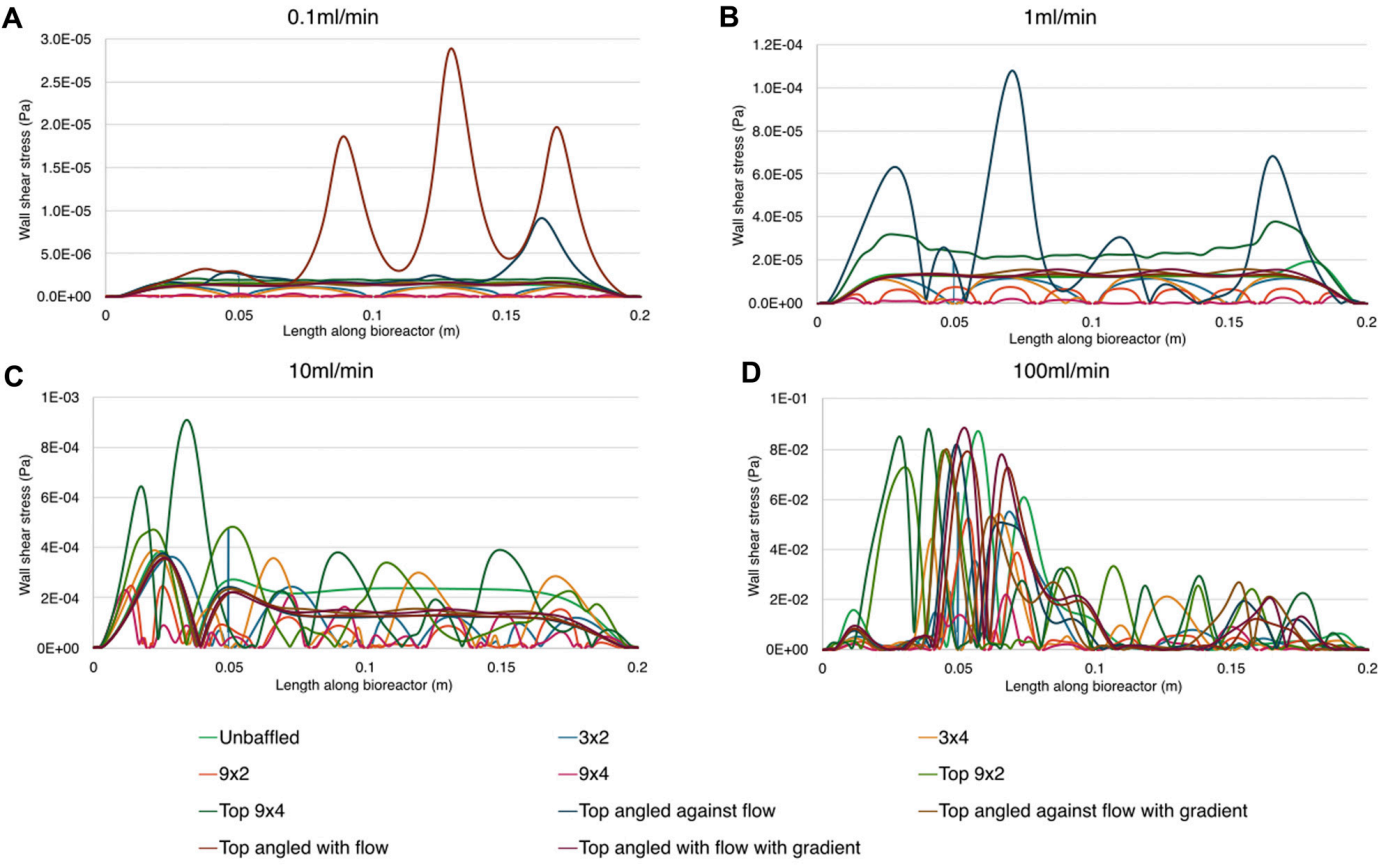
Wall shear stress magnitude along the base of the bioreactor for all nodes for all conditions. Parts show results for different inlet flow rates. **A**: 0.1 ml/min, **B**: 1 ml/min, **C**: 10 ml/min, **D** 100 ml/min.

The inclusion of baffles did indeed influence WSS at the base of the bioreactor, best seen in Figure 8. A 10-fold increase in flow rate resulted in roughly 10× increase in mean WSS between 0.1–10 mL/min, but caused 100-fold increase in mean WSS when transitioning from 10 mL/min to 100 mL/min. While it is not surprising that bottom baffled conditions had lower mean WSS than both unbaffled and top baffled designs, it is interesting that the unbaffled design had lower mean WSS than the top baffled designs at 0.1 mL/min & 1 mL/min flow rates. A possible explanation for this is the effective narrowing of the channel by the top baffles, which resulted in higher mean fluid velocities in accordance with the continuity equation. This higher mean velocity resulted in sharper velocity gradients at the wall, and therefore higher WSS.

### 3.3 Computational models predict negligible risk of cell death due to eddy formation for any bioreactor design

**FIGURE 9:**
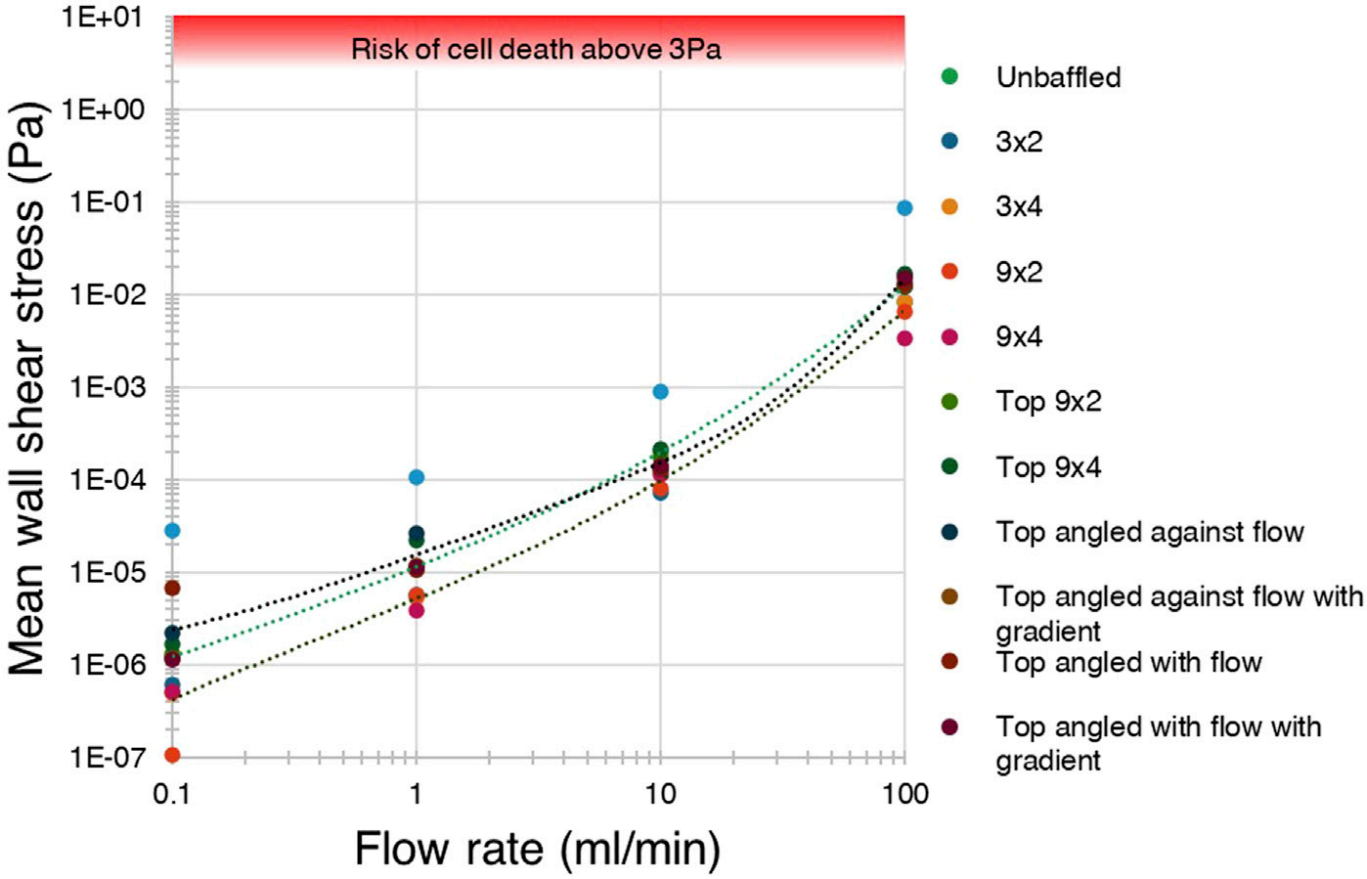
Shear stress was not predicted to exceed the cell-lethal 3 Pa threshold in any condition. A log-log graph of average wall shear stress for each condition against flow rate, showing mean stress did not exceed 0.1 Pa in any condition, and were far below the 3 Pa threshold for cell death.

The mean and maximum WSS generated across all tested bioreactor designs, baffle parameters and flow conditions, were compared with applied flow rates, generating the graphs show in Figure 9 (data included in Supplementary Material). Top baffled conditions produced more wall shear stress on average than the average bottom baffled condition, and slightly more than the unbaffled condition at lower flow rates. A key finding is that, in all cases, neither mean nor maximum shear stresses due to flow and eddies were higher than 0.1 Pa, far below the 30 dyn/cm^2^ generally considered to cause cell death (Moose et al., 2020; Hope et al., 2021).

## 4 Discussion

This study developed an analytical tool to link macro-scale measures of fluid flow to risk of cell death, using established relationships between eddy size and dissipation rates when generated off flat surfaces. Applying this tool computationally to generic bioreactor geometries led to the finding that no condition approaches the shear stresses known to cause cell death, within the eddies. While requiring further biological validation, this provides a novel, accessible and useful tool for bioreactor design, as well as giving confidence that eddy formation off flat surfaces is not a significant risk in typical bioreactors across a range of flow conditions. This approach could also be modified or incorporated to identify risk of cell death through other variables, such as osmotic pressure or dissolved oxygen content.

In all model conditions, wall shear did not peak at levels dangerous to cell health. The shear stress levels found in previous research to be cell-lethal, between 30 and 1,000 dyn/cm^2^ (Moose et al., 2020; Hope et al., 2021), were not reached. At the extreme, a peak wall shear of just 0.88 dyn/cm^2^ was found around the reattachment point in 100 mL/min flow conditions in multiple conditions. Across all flow rates, bottom baffled designs reduced the wall shear stress on the base of the bioreactor when compared to unbaffled or top baffled conditions. This is likely due to flow interruptions reducing velocity between baffles, and hence reducing the velocity gradient between wall and fluid, with similar results found previously in experiments using particle image velocimetry (PIV) (Yoshida et al., 2014).

In general, models tended to exhibit increasingly oscillatory flow and eddy generation as flow rate increased. This is not surprising, as inertial forces dominate viscous forces at higher velocities. An interesting exception was seen in the top angled baffled conditions, which seemed to exhibit more eddy generation in lower flow rates but significantly more laminar flow at medium flow rates. This may indicate that the distance between the bioreactor entrance and first baffle is key to this behaviour. Backward-facing step behaviour (Driver and Seegmiller, 1985) could be seen in the 10 mL/min and 100 mL/min models, where inertial forces dominate, and it is possible the first angled baffle was in a location that disrupted the expected recirculation zone above the jet in the 10 mL/min condition.

The eddy generation observed occurs due to both the baffle inclusion and the inlet jet entraining fluid through viscous effects. The size of the largest eddies appears to be limited to the size of the baffle in low flow rate conditions, half the vessel height in medium flow rate conditions, and the full height of the vessel in high flow rate conditions. Most importantly, no modelled condition produced eddies that are expected to dissipate to cell-scale eddies. In fact, the bioreactors were able to maintain eddies large enough to prevent expected cell death through eddy dissipation in all cases. This is a novel finding not discussed or investigated in literature or industry. This will require experimental validation on varying bioreactor geometries to confirm, particularly as past studies have demonstrated that eddy generation within microcarrier systems can result in cell death in certain circumstances (Sinskey et al., 1981; Hu, 1983; Croughan, 1988). However, if it is true that bioreactor environments do not produce dangerous eddies off flat surfaces at standard flow rates, this knowledge could alleviate the known concerns regarding environmental shear stress that is common in the industry (Papoutsakis, 1991; Millward et al., 1994; Nienow, 2006; Nienow et al., 2013; Chalmers, 2015; Chalmers, 2021).

It should be noted that the computational models used are two-dimensional and steady state, with effect on cells being inferred by measuring flow metrics at nodes. This simplification is for the sake of minimizing computational resource required. Assuming that the cross-section of the bioreactor is constant, and flow is fully developed, the added complexity of a three-dimensional model was not required to initially investigate the proposed relationship between Reynolds number and cell death. Biological validation of this relationship is a recommended priority over adding dimensions *in silico.* It also does not account for any diffusion gradient of dissolved gases or nutrients, which are variables the proposed model relating Reynolds number to cell death does not consider.

Lastly, an interesting method of mixing control is hinted at by these results. By continuously manipulating the flow rate into the vessel, it may be possible to “flip” through fluid behavioural states. For example, a period at 100 mL/min could agitate cells and mix media, refreshing the concentration gradients of nutrients and cell output products, followed by a prolonged period at a lower flow rate, if any flow at all, to allow cells to sediment and expand. If optimised, this behaviour could aid cell expansion by reducing concentration gradients or maintaining appropriate cell density, and also aid in automating quality assurance or quality control processes through the homogenization of bioreactor contents for sampling procedures.

In conclusion, this study developed an analytical tool for researchers and manufacturers to link macro-scale measures of flow to risk of cell death using relationships with eddy size and dissipation rates. Putting this into practice computationally, a generic bioreactor geometry has been modelled with 11 variants to investigate flow behaviour, with eddy measurements taken. No combination of flow condition or design parameter was predicted by the tool to cause cell death within eddies, indicating negligible risk of cell death due to eddy formation off flat surfaces within non-microcarrier cell culture systems. While this requires experimental validation, this tool nonetheless provides reassurance and accessible prediction of bioreactor design parameters that could result in cell death.

## Data Availability

The original contributions presented in the study are included in the article/supplementary material, further inquiries can be directed to the corresponding author.
